# Barriers and motivators to gaining access to smoking cessation services amongst deprived smokers – a qualitative study

**DOI:** 10.1186/1472-6963-6-147

**Published:** 2006-11-06

**Authors:** Elin Roddy, Marilyn Antoniak, John Britton, Andrew Molyneux, Sarah Lewis

**Affiliations:** 1 Division of Respiratory Medicine, School of Medical and Surgical Sciences, University of Nottingham, Nottingham, UK; 2 Division of Epidemiology and Public Health, School of Community Health Sciences, University of Nottingham, Nottingham, UK

## Abstract

**Background:**

Smoking is strongly associated with disadvantage and is an important contributor to inequalities in health. Smoking cessation services have been implemented in the UK targeting disadvantaged smokers, but there is little evidence available on how to design services to attract this priority group.

**Methods:**

We conducted focus groups with 39 smokers aged 21–75 from the most socio-economically deprived areas of Nottingham UK who had made an unsuccessful attempt to quit within the last year without using smoking cessation services, to identify specific barriers or motivators to gaining access to these services.

**Results:**

Barriers to use of existing services related to fear of being judged, fear of failure, a perceived lack of knowledge about existing services, a perception that available interventions – particularly Nicotine Replacement Therapy – are expensive and ineffective, and negative media publicity about bupropion. Participants expressed a preference for a personalised, non-judgemental approach combining counselling with affordable, accessible and effective pharmacological therapies; convenient and flexible timing of service delivery, and the possibility of subsidised complementary therapies.

**Conclusion:**

We conclude that smokers from these deprived areas generally had low awareness of the services available to help them, and misconceptions about their availability and effectiveness. A more personalised approach to promoting services that are non-judgemental, and with free pharmacotherapy and flexible support may encourage more deprived smokers to quit smoking.

## Background

There is a strong relationship between cigarette smoking and social disadvantage. Smoking prevalence and levels of addiction to tobacco are highest in the most disadvantaged social groups [1,2] as are difficulties in quitting due to perceived low self-efficacy [3,4] and triggers for habitual smoking in terms of stressors [5,6]. Despite the fact that deprived smokers are as likely or more likely than advantaged smokers to want to quit smoking [7], smoking prevalence has decreased markedly in the most socially advantaged groups over the past two decades but remained unchanged in the most disadvantaged smokers [1], many of whom could be categorised as 'hard core' smokers [8]. As a result, smoking remains the largest single identified cause of the inequalities in health between rich and poor in the UK.

In 1998, the UK government Tobacco White Paper [9] outlined a strategy to develop smoking cessation services in the National Health Service, based on effective [10] and cost effective [11,12] models, and with particular emphasis on helping disadvantaged smokers. These services have been successful in reaching smokers from disadvantaged communities [13], largely through locating them within areas of greatest deprivation, but otherwise there is little evidence to develop strategies to attract this target group [14]. It is therefore important to explore how services are perceived by those in the target groups to provide insights into how access to and uptake of services could be improved. Nottingham's smoking cessation service, New Leaf, provides a standard range of evidence-based smoking cessation interventions typical of those available nationally[15]. In this study, we have attempted, using qualitative methods, to determine whether disadvantaged smokers in Nottingham are aware of existing local smoking cessation services, to explore how they view the services on offer, and to identify specific barriers and motivators to improve access to smoking cessation services in this target population.

## Methods

We used Manchester Information and Associated Services[16] to identify 5000 households in the 5% most deprived (highest Townsend Score) enumeration districts in Greater Nottingham, and posted a short self-completion questionnaire to these addresses. A second questionnaire and reminder letter was sent to non-respondents six weeks after the first questionnaire. The questionnaire asked how many smokers there were in the home over age 16 and whether each smoker wanted to quit smoking and had made an attempt to quit smoking within the previous 12 months. The questionnaire also asked for contact details of any smokers in the home who would be willing to help further by attending a discussion group. We offered to provide a language translation or help by telephone in completing the questionnaire as appropriate. We used the responses to select purposively participants who were current smokers, living in one of the 5% most deprived enumeration districts in Greater Nottingham, and who had made an attempt to quit smoking within the last year without using local smoking cessation services. Assuming that up to 35% of adults in manual occupations smoke, with an average of 2 adults per household, our questionnaire had the potential to reach around 3,500 smokers, of whom about one third (1167) would have made a quit attempt in the past year [7]. We chose to do this using qualitative methods which, although they have a background in marketing and social research, are increasingly being used in medicine. They aim to provide a holistic perspective which preserves the complexities of human behaviour [17] and explore the ideas and concerns which the subjects themselves come up with [18] rather than imposing external research themes. The study was approved by the Nottingham City Hospital research ethics committee.

Focus groups were held in local premises in the early evening, inviting 10 participants to each session. Each participant was sent a study information sheet in advance of the group session, and at the beginning of each session we also gave a verbal explanation of the aims of the study, along with the assurance that there was no obligation to participate in the research, and obtained signed consent to proceed. The discussions were semi-structured, and facilitators (2 per group) used a discussion guide which covered the following broad topics: participants' smoking behaviour, experience of quitting, knowledge and perceptions of existing smoking cessation services, and characteristics of services that would be more attractive to the smoker. We used a grounded theory approach [19], interweaving data collection and analysis from the start, and refining the topic guide to reflect the emerging themes. We used a variety of visual stimuli to prompt discussion of smoking cessation methods including leaflets, pictures of nicotine replacement therapy, flip chart listing of unprompted cessation methods, and pile-sorting exercises – sorting pictures representing various aids to smoking cessation into 'good' and 'bad' piles. Sessions lasted between 90 and 120 minutes, with breaks as necessary. Focus group sessions were tape recorded and transcribed in full.

We used a group approach to analyse the data in a systematic and rigorous manner, and to minimise bias in interpretation of findings from the text of the transcripts. Initially each researcher (ER, AM & SL) worked independently reading a subsample of three transcripts to identify themes (main issues relevant to smoking cessation). We met to agree these themes and to develop provisional definitions of the main themes, then independently reread the same transcripts to identify key issues or categories within themes, and met once again to agree working definitions of categories within themes. Two researchers (ER & AM) read the remaining transcripts to further develop themes and categories, in an iterative process of refining and developing categories where data did not fit existing definitions [19]. One researcher (ER) then coded all transcripts using agreed definitions, and using NUD*IST 6 software (QSR International, 2000) to facilitate a systematic inspection of text coded under each category. We aimed to describe the themes and the principal variations in opinions and attitudes within them, rather than imposing any predetermined categories. Anonymous quotes from the transcripts have been used to illustrate the themes raised.

## Results

A total of 358 questionnaires were returned with a median Townsend score of 8.1 (interquartile range 7.9 – 8.3). Of those returned, 186 were from smokers, of whom 92 declined or were unsuitable to participate in focus groups, 94 agreed to take part, and met the eligibility criteria, and 39 (median age 45 years, age range 27 – 77 years, 23 males) actually participated. Nine focus groups of sizes 2, 2, 3, 4, 4, 5, 5, 7, 7 participants were carried out.

Most participants had been smoking since their teenage years and many described smoking initiation as a rite of passage at an age when they were unaware of the health risks of smoking. Participants perceived themselves as highly addicted, with cigarettes very much in control of and ingrained into their daily routine (Table 1). Participants used a wide range of justifications for continuing to smoke and raised many concerns about quitting, mostly based on negative experiences in the past (Table 2). Willpower was still considered to be the most important adjunct to quitting by most participants although some others regarded willpower as ineffective based on previous unsuccessful attempts at quitting. Knowledge of access to cigarettes was far greater than knowledge of cessation support available (Table 3) – brand and price were particularly important factors when buying cigarettes, and some participants were open about the purchase of low-price contraband cigarettes. Participants felt marginalised by government policy, highlighting perceived contrasts between services for smokers and those for drug and alcohol users (Table 4). Participants also expressed feelings of victimisation by non-smokers and doctors (Table 4), with a perception that these attitudes permeated smoking cessation services (Table 5), despite the fact that none of the participants had accessed such services.

Barriers to access particularly related to lack of knowledge of services and misconceptions about attitudes and availability (Table 5). Many participants used strong language to describe the intensive and often extreme support that they felt they would need to quit – 'magic wand', 'brain transplant', 'someone watching me all the time', or 'lock me in a room'. Nicotine Replacement Therapy was perceived as expensive and ineffective with many contraindications. Bupropion was also perceived negatively with some participants referring to recent adverse reports in the media, in contrast to complementary therapies which were generally regarded as more effective than pharmacotherapies (Table 6). Respondents felt that publicising cessation services more appropriately to them and offering some form of personalised approach might make them more likely to access them. (Table 7). Some suggested novel approaches to quitting and perceived that peer support or incentives might encourage smokers to access services – these approaches are also summarised in table 7. Most participants were unaware that the local smoking cessation service is already providing many of the services that they considered desirable.

## Discussion

The participants in this study were smokers who had previously attempted to quit smoking without formal support, and who lived in extremely deprived areas. Although motivated to quit smoking, they felt that their smoking was intractable and were torn between thinking that only intensive measures would help them to stop but also that all that was really required was willpower. They felt increasingly marginalised by society and government and felt that their addiction was not taken as seriously as addiction to heroin or alcohol. They knew little about the services available to help them, but perceived them to be ineffective and expensive despite evidence to the contrary. Participants stated the need for a wide variety of cessation group timings and locations without being aware that these services already existed.

This study addresses access and perception of services by deprived smokers, an important public health issue which has been under explored in previous research. To our knowledge, this is the first study that directly addresses barriers and motivators to accessing smoking cessation services amongst the social groups with the greatest need although studies have been carried out with pregnant women [20-22] and smokers also dependent on alcohol [23]. We expected a low response to our questionnaire, and indeed of the estimated 1167 potentially eligible smokers expected among the 5000 households included in our initial questionnaire, we recruited just 39 to take part in focus groups. Those who took part are unlikely to be representative of all eligible smokers in this social group. In particular, they were recruited to the focus groups by means of letters and phone calls, and these may be individuals who were more responsive to a personalised approach. Moreover, focus groups always provide a platform for the more opinionated in each group and despite the best efforts of the facilitators may not be representative of the true views of each individual within a group, and some of the groups were very small. However, the participants were, by definition, motivated smokers whose needs were not being met by current services, and who therefore provided an appropriate context for the current study. That our study was based in an area served by a single smoking cessation service, New Leaf, potentially limits the external validity of our findings. However, New Leaf provides a standard range of evidence-based smoking cessation interventions, with group or individual support at flexible times and locations, typical of the services offered nationally. Moreover, since the New Leaf service is based in one of the original Health Action Zones, offers predominantly individual support and has a strong relationship with local Primary Care Trusts, the reach of the service to disadvantaged smokers is likely to be at least as great as that for services elsewhere in the UK [24]. We suggest therefore that the unmet needs identified for deprived smokers in Nottingham are likely to be applicable more widely.

The main themes of our findings are however supported by evidence from other studies, showing that smokers from areas of disadvantage feel unable to cope without cigarettes [25,26], guilty about their continued smoking [27], are sceptical about the effectiveness of nicotine replacement therapy [5] and less likely to be able to overcome barriers to cessation despite high levels of motivation to quit smoking [26,27]. The perception that smokers from deprived areas are in some way discriminated against [26] and discouraged from making healthy choices is also reported elsewhere [28], as is the easy availability of contraband cigarettes in areas of deprivation [29], which may act as a barrier to cessation. A recent cross sectional survey of adult UK smokers identifies the need for targeted interventions, particularly towards smokers in areas of socio-economic deprivation [8]. The English smoking cessation services were instructed to target this group, as well as young and pregnant smokers, and there is evidence showing that these services have been successful in reaching smokers from disadvantaged communities [13]. However, these services may have been hindered in the need to meet throughput targets and by lack of evidence to develop strategies to attract this priority group [14]. Our findings go some way to identify the needs of these smokers, and specifically to identify factors which act as barriers or motivators to them gaining access to these services.

## Conclusion

Smoking cessation services are amongst the most effective and cost effective interventions available in medicine [10-12], but if they are to deliver their enormous public health potential, particularly in relation to smokers in deprived areas, they need to be more widely and appropriately promoted in an attempt to dispel some of the myths surrounding their availability and effectiveness held by these smokers. A more personalised approach to promoting services that are non-judgemental, and with free pharmacotherapy and flexible support may encourage more deprived smokers to quit smoking.

## Competing interests

The author(s) declare that they have no competing interests.

## Authors' contributions

JRB, SAL and AM conceived the study; MA distributed questionnaires and administered groups; AM, ER, SAL and MA facilitated focus groups; ER, SAL and AM analysed the data and ER wrote the first draft of the paper which was subsequently commented on by all other authors. All authors read and approved the final manuscript.

## Pre-publication history

The pre-publication history for this paper can be accessed here:



## Figures and Tables

**Table 1:** Participants interpretations of smoking experiences:

*Smoking Initiation*
'I was about 10. Didn't realise because in them days, smoking wasn't bad for you, it was considered to be good for you, if you smoked Marlboro, kids wanted to be a cowboy, a tough guy, all my heroes smoked, Bogart, Cagney, all had a fag in their mouth........'
'Smoking was good for you when I started.'
'I think it was when I started down pit, everybody, they'd come up and they had a fag straightaway, I just done the same thing.'
*Smoking behaviour and addiction*
'Most people have a coffee, sit down with a cigarette to get their bearings, you stop to think about what you're going to do in the day ...' by the time you've put the fag out you're ready to go out there and challenge every one, it perks you up a bit.'
'You finish washing up and you have a fag, when you have a cup of tea you have a fag, when you sit down and watch Neighbours you have a fag ...'
'I'll be honest with you now, I'm sitting here now and I'm getting really, really sort of agitated! It's like I can't talk to people normally without a cigarette and I know that I'm talking here, but I'm slowly twiddling, my toes are tapping ...'
'If they gave me £50 m, I still couldn't stop, not for £50 m I couldn't.'
*Who's in control, you or the fags? *'The fags, definitely the fags.'

**Table 2:** Participants experiences of smoking cessation attempts

*Justification for continued smoking*
'It's the stress factors, you're stressed out because you smoke so you smoke more.'
'There are people that you do know who have never touched a cigarette and they die of cancer.'
'You're going to die whether you smoke or not.'
*Concerns about quitting smoking/previous quit attempts*
'The mental obsession is there all the time .....you need a fag, you need a fag, want a fag.'
'The first week was terrible because I got really violent .. ..'
'...I know when I stop smoking the weight goes on.'
'You go and fetch the paper.....you buy ten fags and hide them because you don't want her to think that you've cracked, you haven't really cracked because you haven't even started, so you're lying to yourself at the start.'
'You keep trying and trying, but you just get fed up because you know what's coming don't you?'
*Willpower*
'I know all the statistics, I know all the stuff that's there to know and I'm a strong willed person, anything else I've overcome in my life, massive problems and all the rest of it, but one of the things has been a constant through my life is the smoking, it's that element of .... having tried and failed.'
'......okay, it sounds a bit crude but if I've got diarrhoea, all the willpower in the world doesn't stop me from running to the toilet, that's like willpower, I can be determined not to smoke again and I just make myself a failure, and it's not even my failure, it's everybody else outside saying ....you're a weak person for not being able to do this.'

**Table 3:** Access to cigarettes

'...cigarettes are everywhere, the shelves are full of them, so even if you're giving up, you walk into the supermarket, everyone around you smokes, wherever you go.'
'This is the thing, if you're sat in a pub and somebody says, "I've got some fags here", and they're £2.50 or £3 and you've got to (normally) pay £4 odd, you're going to take them aren't you?'
'I wouldn't buy so many I don't suppose if I'd got to pay full price.'

**Table 4:** Participants interpretation of the attitudes of others to smokers

*Attitudes of society (non-smokers) and government*
'They look down on you ...get funny looks, like you've committed a crime.'
'I sometimes think we're ...... pariahs because we smoke.'
'I remember I was waiting for a bus, I lit a cigarette up, there was a child there of about 5 or 6 years with his Mum, the child goes, "look what that man's doing Mummy", I thought "ergh".'
'....it's non smokers, they're like Born Again Christians, they go on a witch hunt, if they know you smoke'
'It's an addiction, I bet drug users get free stuff ....why don't they give us free stuff?'
'You've got Alcoholics Anonymous, but they don't think about smokers.'
'The Government doesn't want to stop it because of the amount of money they get from the whole country smoking.'
*Attitudes of doctors*
'I was rushed into hospital with a suspected heart attack, he stood at the bottom of the bed, "do you smoke, well stop it".'
'He said "I'm not going to nag at you, count it not as a failure because you went those 12 months, but just keep remembering how good you felt in those 12 months" and he went onto explain and said
"go away and think about it, if you ever want to come in...". But the lady (doctor) has never, ever offered me any sort of help just "how many do you smoke?" ....she just upsets me so much ...'

**Table 5:** Participants perceptions of smoking cessation services

'... we're reasonable intelligent people, we know all the pitfalls, I would like some sort of encouragement or support while packing it in, but it's all the other bit that you have to put up with so that puts you off a little bit, the information bit about it, it puts you off because we've heard it all before'
'To be honest with you I find the more they preach to you about what it does to you, the more I seem to smoke. I know I shouldn't but you just feel that you need one ...'
'You can have far too much of it, and you think "blow it", you let it go in one ear and out the other.'
'I don't know if they'd have me back (if I failed), I don't know if you get second chances with them or third chances!'
'I need some help but you don't know where to go with smoking, because it's legal, you don't know where to go for help and where to ask'
'I don't think I've heard of any (cessation services) to be honest.'
'I can think of lots of people who would like to give up smoking and they've not had a single bit of help and they can't afford to smoke or anything ...'
'They don't know where to go or what to do to get help, they hear about it but the daily business (of living) ... carries on'
'... it's really awkward because you've got Mums with young children who can't get out in the evening, then you've got workers who can't get out during the day because you work.'

**Table 6:** Participants perceptions of smoking cessation therapies

*Nicotine Replacement Therapy*
'I went to the doctors and said, "I want to pack it up", she was looking through the book, says "you can't have the patches, you can't have the chewing gum, you can't have this .." I said "how the hell am I going to stop?".'
'I've heard a few things about the patches, I've heard that they're not much good.'
'About a week I tried, I tried them patches, the inhalers and the chewing gum, but it's the cost of them really, I would have carried it on if it weren't for the cost.'
'For £20, once you've opened it and used it that's it.'
'Cheaper to buy a packet of fags.'
'They're looking at it this way, if you can afford to smoke, you can afford to buy the patches and that's the wrong attitude because blow that for a lark, I'm not paying that sort of money to give up something that I want to do.'
'....they need to find something .....that is actually is as strong as the nicotine in cigarettes, if someone can find another fix that's as strong as the one we're already getting, then that will work.'
*Bupropion*
'I think if it hadn't have been for that outcry about the Zyban, I might have carried on with it but I just left it and that was it.'
'I don't know, if they want me to try that Zyban or something then I'd be a bit scared to take them as well, people have died from taking them or connected to it.'
'I've heard it's got bad side effects ...Someone had strokes on it.'
*Complementary therapies*
'I had a friend of mine and he used to smoke 40 a day, he went on that hypnosis, and from that day on he never smoked a cigarette.'
**'**I'd like something like hypnosis or anything to make you forget that you're addicted to it ...'

**Table 7:** Participants suggestions for novel approaches to promoting smoking cessation

'I think it's you have to advertise that you're giving something..... if you give somebody a 10 p voucher for a can of beans, they'll buy it because it's a voucher.'
'You need something to zap it up, like "it could be you", something to grab your attention.'
'So maybe it should say a bit more, like you say, people like a bargain.'
'I think everything to do with junkies should be designed personally, we're all very, very different.'
'It was addressed to me personally that made me come (to the focus group), I thought "how did they know I smoke?", and I thought "I'll have a look at this, this is interesting"....'
'Small localised groups, I think that's a good idea because there's a chance you'll bump into someone on the street who's going through the thing you're doing, you start to form networks'
'.... if every time you smoked a cigarette you got a purple mark on top or your teeth, you know like that stuff where you don't clean your teeth properly when you were a kid ...'
'...... a voluntary visit to people in the hospitals who are dying through smoking.'
'If you saw directors of tobacco companies and they weren't smoking, they wouldn't let their kids smoke, if you saw the lifestyle that we pay for them to live in, maybe that would work ...'
